# Genome-wide identification and characterization of long non-coding RNAs in developmental skeletal muscle of fetal goat

**DOI:** 10.1186/s12864-016-3009-3

**Published:** 2016-08-22

**Authors:** Siyuan Zhan, Yao Dong, Wei Zhao, Jiazhong Guo, Tao Zhong, Linjie Wang, Li Li, Hongping Zhang

**Affiliations:** Farm Animal Genetic Resources Exploration and Innovation Key Laboratory of Sichuan Province, Sichuan Agricultural University, Chengdu, 611130 China

**Keywords:** Muscle development, LncRNA, Goat, Transcriptome, *cis*-acting, *trans*-acting, Differential expression

## Abstract

**Background:**

Long non-coding RNAs (lncRNAs) have been studied extensively over the past few years. Large numbers of lncRNAs have been identified in mouse, rat, and human, and some of them have been shown to play important roles in muscle development and myogenesis. However, there are few reports on the characterization of lncRNAs covering all the development stages of skeletal muscle in livestock.

**Results:**

RNA libraries constructed from developing *longissimus dorsi* muscle of fetal (45, 60, and 105 days of gestation) and postnatal (3 days after birth) goat (*Capra hircus*) were sequenced. A total of 1,034,049,894 clean reads were generated. Among them, 3981 lncRNA transcripts corresponding to 2739 lncRNA genes were identified, including 3515 intergenic lncRNAs and 466 anti-sense lncRNAs. Notably, in pairwise comparisons between the libraries of skeletal muscle at the different development stages, a total of 577 transcripts were differentially expressed (*P* < 0.05) which were validated by qPCR using randomly selected six lncRNA genes. The identified goat lncRNAs shared some characteristics, such as fewer exons and shorter length, with the lncRNAs in other mammals. We also found 1153 lncRNAs genes were neighbored 1455 protein-coding genes (<10 kb upstream and downstream) and functionally enriched in transcriptional regulation and development-related processes, indicating they may be in *cis*-regulatory relationships. Additionally, Pearson’s correlation coefficients of co-expression levels suggested 1737 lncRNAs and 19,422 mRNAs were possibly in *trans*-regulatory relationships (*r* > 0.95 or *r* < −0.95). These co-expressed mRNAs were enriched in development-related biological processes such as muscle system processes, regulation of cell growth, muscle cell development, regulation of transcription, and embryonic morphogenesis.

**Conclusions:**

This study provides a catalog of goat muscle-related lncRNAs, and will contribute to a fuller understanding of the molecular mechanism underpinning muscle development in mammals.

**Electronic supplementary material:**

The online version of this article (doi:10.1186/s12864-016-3009-3) contains supplementary material, which is available to authorized users.

## Background

Genome-wide transcriptional studies have revealed that large regions of eukaryotic genomes are transcribed into a heterogeneous population of non-coding RNAs (ncRNAs). Generally, ncRNAs shorter than 200 nucleotides are usually described as small/short ncRNA, such as microRNAs (miRNAs), PIWI-interacting RNAs (piRNAs), small interfering RNAs (siRNAs), and classical ncRNAs such as ribosomal RNAs (rRNAs), transfer RNAs (tRNAs), and small nucleolar RNAs (snoRNAs), whereas ncRNAs longer than 200 nucleotides are described as long ncRNAs (lncRNAs). In the past few years, an increasing number of lncRNAs have been discovered in mammal, including *Homo sapiens* [1, 2], *Mus musculus* [3–7], *Bos taurus* [8, 9], *Sus scrofa* [10–13], and *Ovis aries* [14]. And accordingly unveiled that lncRNAs play critical roles in biological processes like transcriptional regulation [15–17], epigenetic modification [18–20], development [21–23], cell differentiation [24–26], as well as in some diseases [27–29].

As important economic animals worldwide, domestic goats (*Capra hircus*) are raised mainly for meat production. Thus, unveiling the molecular mechanisms underneath skeletal muscle formation and development is of vital interest. Muscle development is a complex process that requires the concerted expression and interaction of multiple factors [30]. Several recent studies have indicated that lncRNAs play crucial roles in myogenic differentiation and myogenesis [31–35]. Nevertheless, currently the majority strategy for exploring molecular mechanisms underlying skeletal muscle growth and development in mammals [36–38] is detecting the expression and functions of coding genes like the MRF (myogenic regulatory factor) [39, 40], MEF2 (myocyte enhancer factor-2) [41, 42] families, and the paired box proteins [43], though high-throughput sequencing technologies is also employed to profile expression of miRNA and mRNA expression in goat [44, 45]. Therefore, information about skeletal muscle development-related lncRNAs is still limited especially in goats.

Here, we report the systematic identification and characterization of lncRNAs in fetal and postnatal goat skeletal muscle using an Illumina HiSeq 2500 platform. A total of 3981 lncRNA transcripts were identified and 577 of these transcripts were significantly differentially expressed in pairwise comparisons between RNA libraries of skeletal muscle at the different development stages. To the best of our knowledge, no other report on muscle lncRNAs and their biological functions in goat is currently available. Our results will provide a useful resource for better understanding the regulatory functions of lncRNAs in goat and for annotating the goat genome, as well as contribute to better comprehending skeletal muscle development in mammals.

## Results

### Overview of RNA sequencing (RNA-seq)

To identify lncRNAs expressed in goat skeletal muscle development, we constructed 11 cDNA libraries (E45-1, E45-2, E45-3, E60-1, E60-2, E105-1, E105-2, E105-3, B3-1, B3-2, B3-3) from goat *longissimus dorsi* muscle samples at four developmental stages: three gestation stages at 45, 60, and 105 days of gestation (E45, E60, and E105), and one postnatal stage (B3). Three biological replicates for E45, E105, and B3, and two biological replicates for E60 were used. The libraries were sequenced using an Illumina HiSeq 2500 platform and 125 bp paired-end reads were generated. A total of 1,052,994,828 raw reads were generated in all 11 libraries. After discarding adaptor sequences and low-quality reads, we obtained 1,034,049,894 clean reads. The percentage of clean reads in each library ranged from 97.90 to 98.53 % (for details of the sequencing results see Additional file 1). We mapped the clean reads to the goat reference genome sequence (CHIR_1.0, NCBI). Approximately 75.20–86.60 % of the clean reads in all the libraries were mapped to the goat reference genome (Additional file 1). The mapped sequences in each library were assembled and a total of 56,710 unique assembled transcripts were obtained.

### Identification of lncRNAs in goat skeletal muscle

**Fig. 1:**
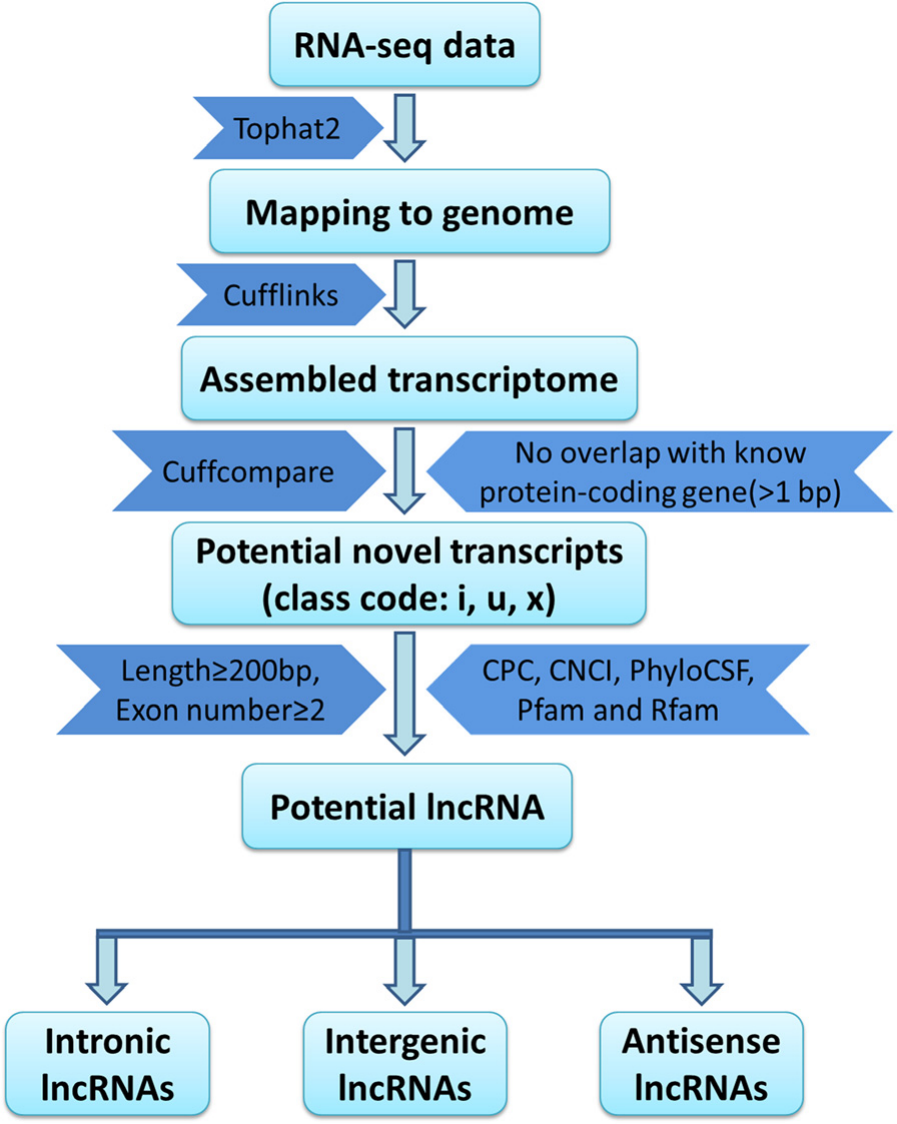
Identification pipeline for lncRNAs. Each step is described in detail in the Methods section

**Fig. 2:**
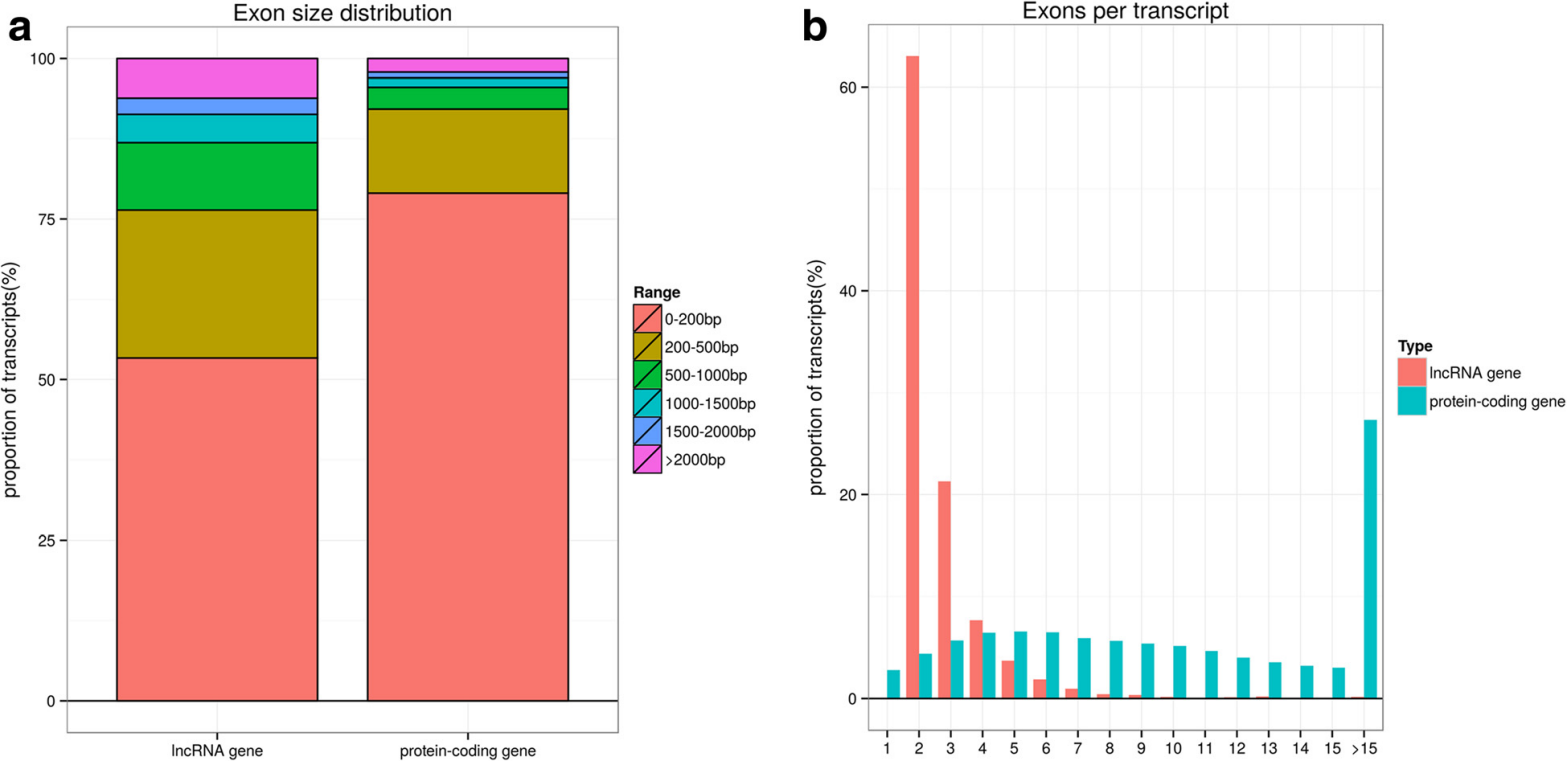
Comparison of the features of goat lncRNAs and protein-coding genes. **a** Exon size distribution of goat lncRNAs and protein-coding genes. **b** Exon numbers per transcript of goat lncRNAs and protein-coding genes

We developed a highly stringent filtering pipeline to discard transcripts that did not have all the characteristics of lncRNAs (Fig. 1). Our pipeline yielded 3981 lncRNA transcripts, including 3515 intergenic lncRNAs (88.29 %) and 466 anti-sense lncRNAs (11.71 %) (Additional file 2), These transcripts corresponded to 2739 lncRNA genes, an average of 1.5 transcripts per lncRNA locus. We found that the lncRNA transcripts were distributed in all chromosomes except the Y chromosome (Additional file 3). The Illumina RNA-seq also produced 24,383 protein-coding transcripts with an average length of 1978 bp and 8.4 exons, which was longer than the lncRNA genes, which averaged 1296 bp in length and 2.4 exons. However, the exon size in the protein-coding genes was smaller than the exon size in the lncRNA genes (most of protein-coding genes were within 200 bp) (Fig. 2a). We also found that protein transcripts with two and three exons accounted for 10.6 % of all the protein-coding genes, which was much lower than the percentage of lncRNA genes with two and three exons (Fig. 2b).

### Identification of differentially expressed lncRNAs

**Fig. 3:**
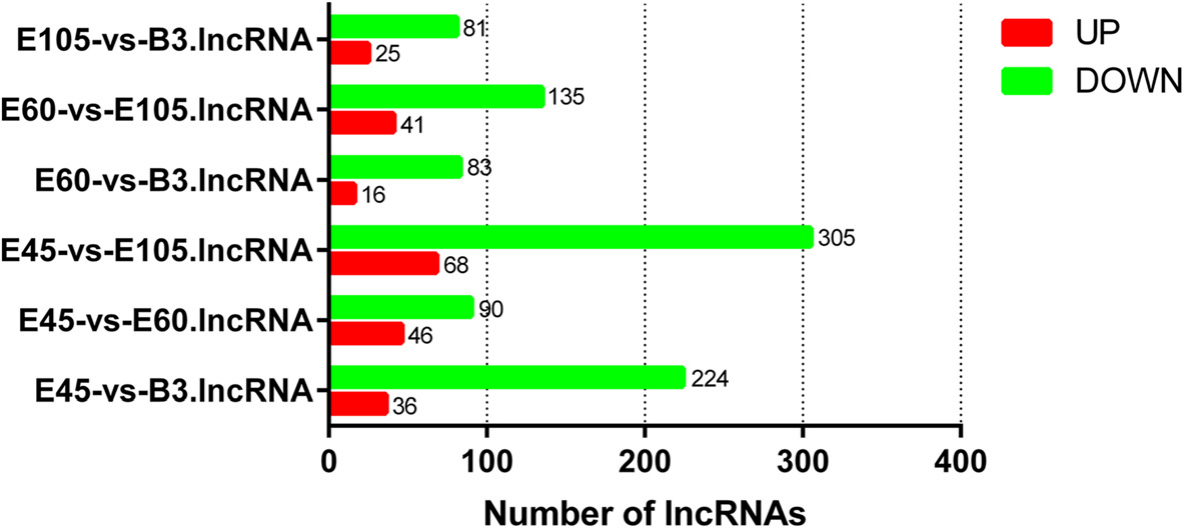
Numbers of up-regulated and down-regulated lncRNAs in goat skeletal muscle at four developmental stages

**Fig. 4:**
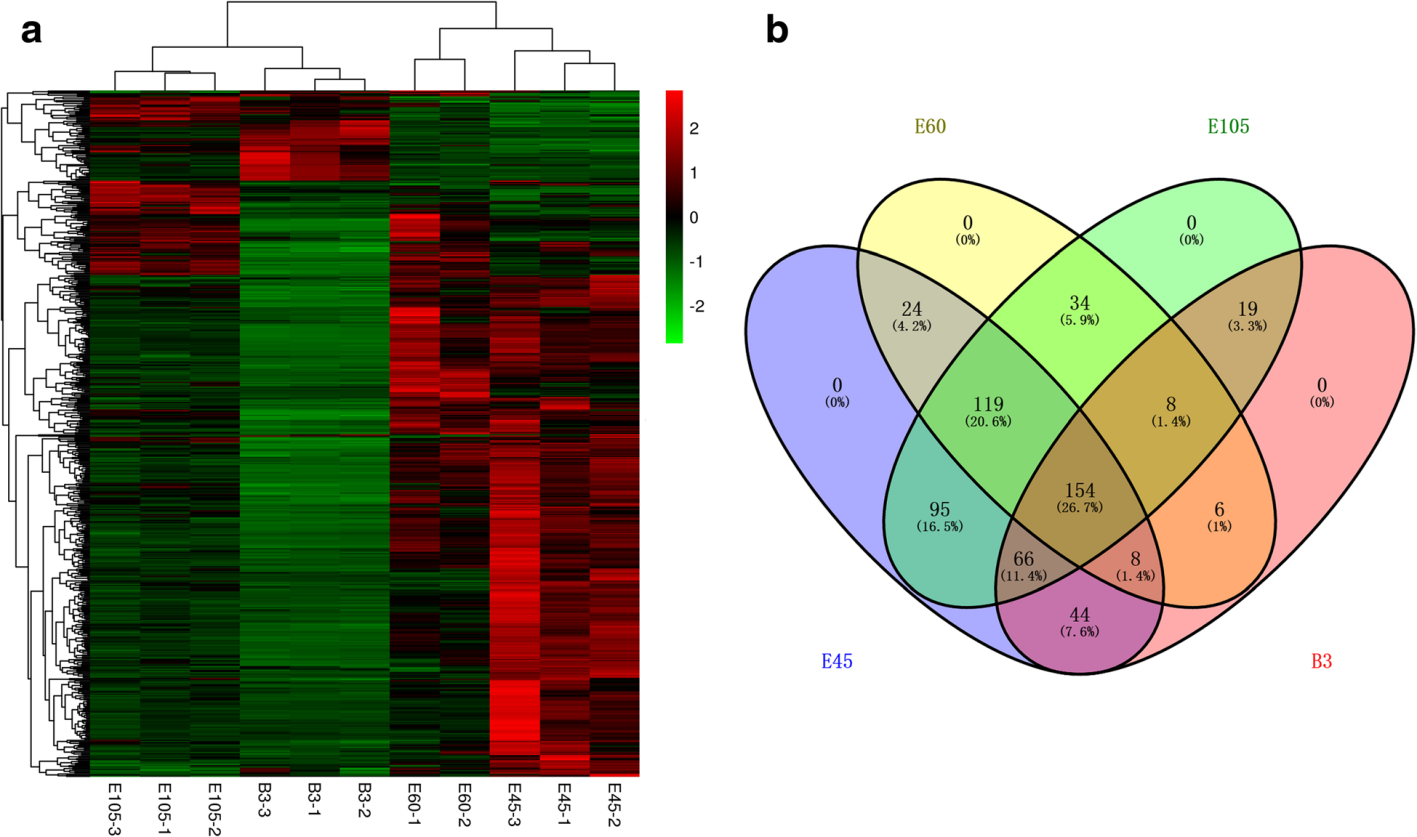
Analyses of differentially expressed lncRNAs in the RNA-seq libraries. **a** Hierarchical clustering analysis of lncRNA expression profiles from 11 libraries with 577 differentially expressed lncRNAs. Data are expressed as FPKM. *Red*: relatively high expression; *Green*: relatively low expression. **b** Venn diagram showing the differentially expressed lncRNAs at the four developmental stages

The expression levels of the lncRNA transcripts were estimated by FPKM (fragments per kilo-base of exon per million fragments mapped) using Cuffdiff. We identified 577 lncRNA transcripts that were differentially expressed during muscle development (Fig. 3 and Additional file 4); the number of down-regulated lncRNAs was higher than the number of up-regulated lncRNAs during development. The expression patterns of differentially expressed lncRNAs were measured by systematic cluster analysis, to explore the similarities and to compare the relationships between the different libraries (Fig. 4a and additional file 5). The replicates for each developmental stage clustered together, and E45 and E60 formed one group and E105 and B3 formed another group. To further analyze the interactions among the differentially expressed lncRNAs, we constructed a Venn diagram using the 510, 353, 495, and 435 lncRNAs that were differentially expressed in E45, E60, E105, and B3, respectively. We did not detect any stage-specific differentially expressed lncRNAs among the four developmental stages, but we identified 154 differentially expressed lncRNAs that were detected in all four developmental stages (Fig. 4b).

### Enrichment analysis of nearest neighbor genes of the lncRNAs

To investigate the possible functions of the lncRNAs, we predicted the potential targets of lncRNAs in *cis*-regulatory relationships. We searched for protein-coding genes 10-kb upstream and downstream of all the identified lncRNAs. We found 1153 lncRNAs that were transcribed close to (<10 kb) 1455 protein-coding neighbors (Additional file 6). Gene Ontology (GO) [46] analysis of the *cis* lncRNA targets was performed to explore their functions. We found 88 GO terms that were significantly enriched (*P* < 0.05), and 12 of these terms were associated with regulation of gene expression. For example, the top 10 enriched terms included nucleotide binding, regulation of RNA metabolic process, DNA-dependent regulation of transcription, transcription regulator activity, and transcription factor activity (Additional file 7). These results suggest that one of the principal roles of lncRNAs may be transcriptional regulation of gene expression. Interestingly, we also found genes, including *RBP4*, *PLN*, *MYLK2*, *RARA*, *CACNB4*, *NR2F2*, *CDK5* and *PITX1*, that were annotated with muscle development-related GO term, striated muscle tissue development (GO:0014706). These results suggest that muscle development may be regulated by the action of lncRNAs on these neighboring protein-coding genes. Pathway analysis [47] showed that the 1547 candidate *cis* target genes were enriched in 252 Kyoto Encyclopedia of Genes and Genomes (KEGG) pathways, several of which were related to muscle development such as insulin signaling pathway, MAPK signaling pathway, TGF-beta signaling pathway, and PPAR signaling pathway (Additional file 7).

### Enrichment analysis of co-expressed genes of lncRNAs

We also predicted the potential targets of lncRNAs in *trans*-regulatory relationships using co-expression analysis. A total of 288,020 interaction relationships (285,161 positive and 2859 negative correlations) were detected between 1747 lncRNA transcripts and 19,846 protein-coding transcripts that corresponded to 7718 protein-coding genes in the goat reference genome (Additional file 8). Functional analysis showed that the co-expressed genes were enriched in 446 GO terms (253 under biological process, 91 GO under cellular component, and 102 under molecular function) that encompassed a variety of biological processes (Additional file 9). Importantly, some of the terms were muscle development-related terms, including muscle cell development (GO:0055001), striated muscle cell development (GO:0055002), muscle contraction (GO:0006936), and muscle system process (GO:0003012). In addition, the co-expressed genes were enriched in 285 KEGG pathways, several of which were related to muscle development, including TGF-beta signaling, MAPK signaling, and PPAR signaling pathways (Additional file 9). These findings indicate that lncRNAs also regulate *trans* target genes.

### Validation of differentially expressed lncRNAs

**Fig. 5:**
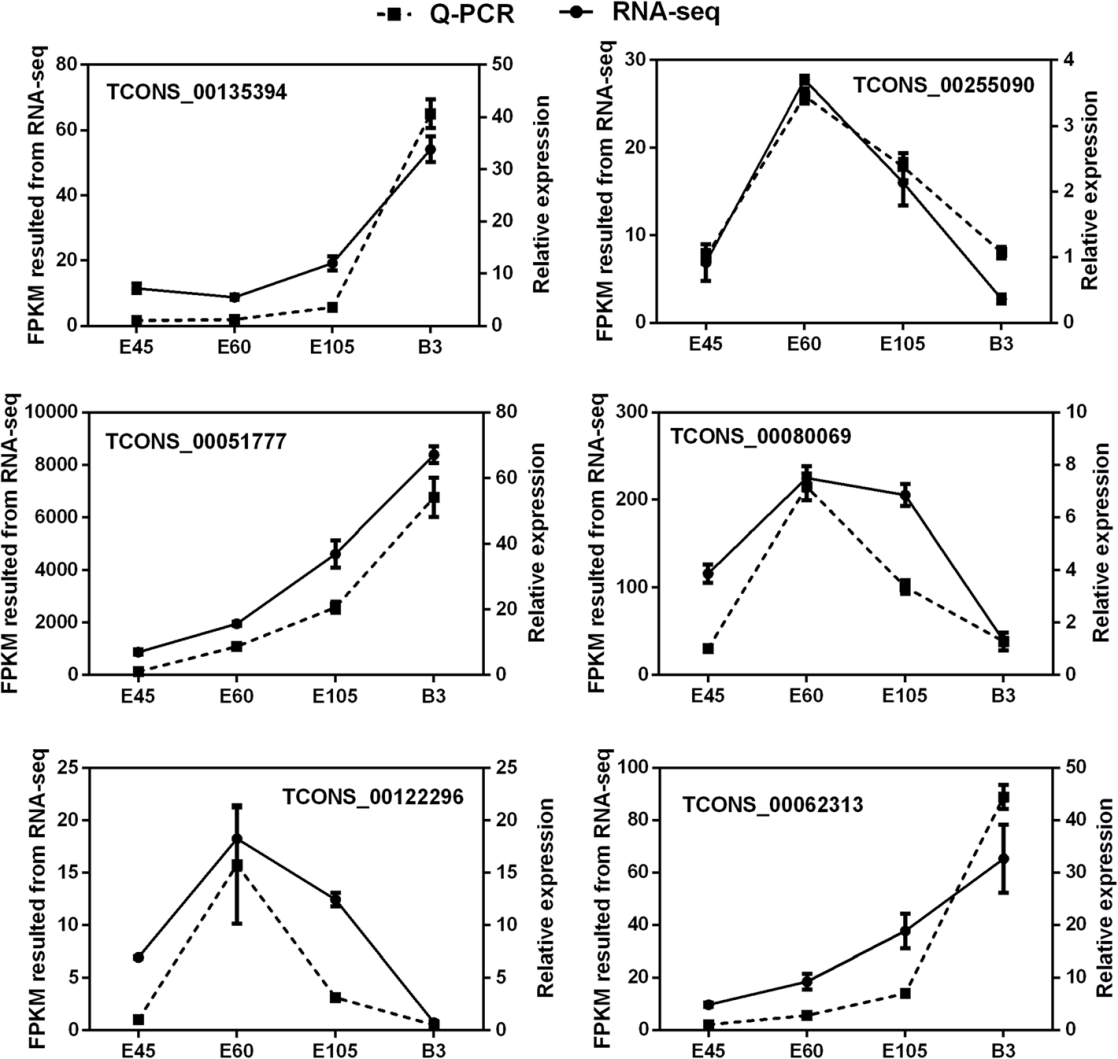
Validation of six differentially expressed lncRNAs by qPCR. Data are the mean ± SEM

We randomly selected six differentially expressed lncRNAs and examined their expression patterns at four developmental stages by qPCR. The results confirmed that the six lncRNAs were expressed at all four development stages (Fig. 5) and showed differential expression at different stages. In addition, the qPCR confirmed that the expression patterns of the six lncRNAs were consistent with their expression levels calculated from the RNA-seq data. All our results show that our pipeline was highly strict in identifying putative lncRNAs, and indicate that most of the identified lncRNAs were truly expressed in vivo.

## Discussion

The identification and characterization of goat lncRNAs, particularly in fetal skeletal muscle development, have been very limited compared with lncRNAs in human [2, 48] and other model organisms [3, 49]. In goat skeletal muscle, the main focus has been on genes and miRNAs rather than on lncRNAs [44, 45, 50, 51]. In the present study, we identified a total of 3981 multiple exon lncRNAs in fetal and postnatal goat skeletal muscle. To the best of our knowledge, this is the first report to systematically identify lncRNAs from RNA-seq data during goat skeletal muscle development.

Non-coding and protein-coding genes are distinguished by their potential coding capability. In this study, we developed a highly stringent filtering pipeline (Fig. 1) to minimize the selection of false positive lncRNAs, which aimed to remove transcripts with evidence of protein-coding potential. We identified 3981 putative lncRNAs with high confidence across four development stages of goat skeletal muscle. In agreement with similar studies on different organisms, the identified putative lncRNAs had fewer exon numbers, shorter transcript lengths, and lower expression levels than protein-coding genes [11, 48, 49]. The number of putative lncRNAs detected in this study was more than that reported in previous studies in cattle and goat [9, 52]. Six randomly selected differentially expressed lncRNA transcripts were validated using qPCR, and the results were consistent with the results from the RNA-seq data. Together, these results confirmed that the identified lncRNAs were of high quality.

LncRNAs are a group of endogenous RNAs that function as regulators of gene expression, and are involved in developmental and physiological processes [23, 53, 54]. We detected 577 putative lncRNAs that were differentially expressed in pairwise comparisons between goat skeletal muscle at the different development stages. These lncRNAs may have specific biological roles in early muscle development in fetal goat. Skeletal muscle development from the fetal to the adult stage consists of a series of exquisitely regulated and orchestrated changes in the expression of many genes [55]. In recent years, the roles of some lncRNAs in muscle biology have been reported. For example, the long intergenic ncRNA muscle differentiation linc-MD1 was the first muscle-specific lncRNA to be identified [24]. Linc-MD1 is required for appropriate muscle differentiation, at least in part because it regulates the levels of myocyte enhancer factor 2C (MEF2C) and mastermind-like protein 1 (MAML1) by sponging endogenous miR-133 and miR-135 in the cytoplasm of muscle cells [24]. In addition, the substantial disintegration of linc-MD1 in primary myoblasts of patients with Duchenne muscular dystrophy suggests that it is likely involved in the pathogenesis of this muscle disorder [24]. Another study revealed that the lncRNA, lncMyoD, regulates skeletal muscle differentiation by blocking IMP2-mediated mRNA translation [56]. Therefore, the differentially expressed lncRNAs reported here can be considered as important novel regulators of goat skeletal muscle biology.

Unlike miRNAs or proteins, the functions of ncRNAs cannot currently be inferred from their sequence or structure; therefore, in this study, we predicted the potential function of the detected lncRNAs using *cis* and *trans* methods. The *cis* nature of a lncRNA refers to its ability to act on a neighboring gene on the same allele from which it is transcribed; thus, this type of lncRNA commonly forms a feedback loop that regulates itself and its neighboring genes.

In the *cis* prediction, we searched for coding genes 10-kb upstream and downstream of all the identified lncRNAs. GO and KEGG analyses of the neighboring protein-coding genes revealed that major enriched pathways were associated with transition metal ion binding, nucleotide binding, zinc ion binding, regulation of RNA metabolic process, regulation of transcription, and transcription regulator activity. These results indicate the possible role of lncRNAs in transcriptional regulation of gene expression. Interestingly, we found some of the *cis* target protein-coding genes were involved in skeletal muscle tissue development (e.g., *MYLK2*, *NR2F2*, *CDK5* and *PITX1*) (Additional file 6), implying that the corresponding lncRNAs play regulatory roles in skeletal muscle development. Several recent studies also indicated that lncRNAs were involved in *cis*-regulatory activity in muscle development; for example, the lncRNA Dum (developmental pluripotency-associated 2 (Dppa2) upstream binding muscle lncRNA) was identified in skeletal myoblast cells [31]. Dum promotes myoblast differentiation and damage-induced muscle regeneration by silencing its neighboring gene, Dppa2, in *cis* through recruiting Dnmt1, Dnmt3a, and Dnmt3b [31]. Similarly, a ChIP-seq study of the Yin Yang 1 (YY1) transcription factor, an important component of the PcG complex that negatively regulates myogenesis, identified a number of lncRNAs regulated by YY1 (YY1-associated muscle lncRNAs or Yams) [57]. Among the Yams, Yam-1 displayed a *cis* effect on the expression of neighboring genes, including one that encodes miR-715, which targets and represses Wnt7b in skeletal muscle.

Many lncRNAs can also function in *trans* mode to target gene loci distant from where the lncRNAs are transcribed [58]. In the co-expression analysis, we detected 1747 lncRNA transcripts that were related to protein-coding genes based on the expression correlation coefficient (*r* > 0.95 or < −0.95). GO enrichment analysis found that the enriched GO terms referred mainly to development process, transcriptional regulation, and biosynthetic process. Furthermore, a cluster of lncRNAs in the co-expression analysis often targeted protein-coding genes that were expressed specifically in muscle and were involved in muscle development (e.g., *TNNT1*, *TNNT3*, *MYH1*, *MYH2*, *MyoG*, and *MYL3*). This is an interesting observation, which indicates the functional complexity of lncRNAs and is worth investigating further. Mousavi et al. [59] found two lncRNAs in two enhancer regions of the *MyoD* gene that they named ^DDR^RNA and ^CE^RNA, where DRR indicates distal regulatory regions and CE indicates core enhancer. The study showed that ^CE^RNA facilitated the occupancy of RNA polymerase II in *cis* by increasing chromatin accessibility, stimulating the expression of *MyoD*, while ^DDR^RNA functions in *trans* to promote the expression of myogenin, a key member of the myogenic transcription factor family. More recently, Mueller et al. [32] identified a lncRNA transcribed upstream of *MyoD* named MUNC (MyoD upstream non-coding RNA), and demonstrated that one of the spliced isoforms of MUNC was ^DRR^RNA. Consistent with the results of Mousavi et al. [59], experimentally decreasing MUNC expression blocked myoblast differentiation, further highlighting the role of enhancer-associated lncRNAs during myogenesis [32]. These results suggest that lncRNAs exhibit regulatory function through *cis*-acting or *trans*-acting mechanisms in skeletal muscle biology and diseases.

All the studies mentioned above have demonstrated that lncRNAs are an integral part of the regulatory network of muscle biology. The present study provides evidence for the role of lncRNAs in skeletal muscle development in goat, which is a starting point for understanding the regulatory mechanisms in which they are involved. The identification of the lncRNAs has greatly improved the annotation of the goat reference genome. Further, we believe that the putative lncRNAs may contribute to a better understanding of the biological basis of regulatory interactions amongst mRNA, miRNA, and lncRNA.

## Conclusions

We elucidated skeletal muscle lncRNA profiles of fetal and postnatal goats by RNA-seq analysis and identified and characterized caprine lncRNAs that may be involved in skeletal muscle development in goat. This study provides a catalog of goat muscle lncRNAs that will help in understanding their regulatory roles in goat muscle development. In future studies, we plan to investigate the functions of some of these lncRNAs to provide basic information that will add to the understanding of the regulatory mechanisms associated with goat muscle development at the molecular level.

## Methods

### Ethics statement

The methods used in this study were performed in accordance with the guidelines of Good Experimental Practices adopted by the Institute of Animal Science (Sichuan Agricultural University, Chengdu, China). All surgical procedures involving goats were performed according to the approved protocols of the Biological Studies Animal Care and Use Committee, Sichuan Province, China.

### Animal and tissue preparation

Jianzhou big-eared goats were used in this study. Three pregnant ewes at each developmental stage were subjected to caesarean section to collect the fetuses at 45, 60, 105 days of gestation, and three female lambs were collected at the third day after birth. *Longissimus dorsi* muscle samples were collected at these four developmental stages: three gestation stages (E45, E60, and E105) and one postnatal stage (B3). Three biological replicates for E45, E105, and B3, and two biological replicates for E60 were collected. The eleven samples were immediately frozen in liquid nitrogen for RNA extraction.

### RNA extraction, library construction, and sequencing

Total RNA was isolated from the 11 libraries using TRIzol reagent (Invitrogen, Carlsbad, CA, USA) according to the manufacturer’s instructions. RNA degradation and contamination were monitored on 1 % agarose gels. RNA purity was checked using a NanoPhotometer® spectrophotometer (IMPLEN, Los Angeles, CA, USA). RNA concentration was measured using a Qubit® RNA Assay Kit in a Qubit® 2.0 Fluorometer (Life Technologies, Carlsbad, CA, USA). RNA integrity was assessed using a RNA Nano 6000 Assay Kit in a Bioanalyzer 2100 system (Agilent Technologies, Santa Clara, CA, USA). Only samples that had RNA Integrity Number (RIN) scores > 8 were used for sequencing. A total of 3 μg RNA per sample was used as input material for RNA sample preparation.

First, rRNA was removed using an Epicentre Ribo-zero rRNA Removal Kit (Epicentre, Madison, WI, USA), and the rRNA-free residue was obtained by ethanol precipitation. Subsequently, high strand-specificity libraries were generated using the rRNA-depleted RNA and a NEBNext Ultra Directional RNA Library Prep Kit for Illumina (NEB, Ipswich, MA, USA) following the manufacturer’s recommendations. Briefly, the rRNA-depleted RNA was fragmented using divalent cations under elevated temperature in NEBNext. First-strand cDNA was synthesized using random hexamer primers and M-MuLV reverse transcriptase (RNase H^−^). Subsequently, second-strand cDNA synthesis was performed using second-strand synthesis reaction buffer, DNA polymerase I, and RNase H. Remaining overhangs were converted into blunt ends by exonuclease/polymerase activity. After adenylation of the 3’ ends of the DNA fragments, NEBNext adaptors with hairpin loop structures were ligated to the fragments to prepare them for hybridization. To select cDNA fragments that are 150–200 bp in length, the fragments in each of the library were purified with an AMPure XP system (Beckman Coulter, Brea, CA, USA). Then 3 μl USER Enzyme (NEB, Ipswich, MA, USA) was used with size-selected, adaptor-ligated cDNA at 37 °C for 15 min followed by 5 min at 95 °C before PCR. The qPCRs were performed with Phusion High-Fidelity DNA polymerase, Universal PCR primers, and Index (X) Primer. The PCR products were purified (AMPure XP system) and library quality was assessed on an Agilent Bioanalyzer 2100 system. Clustering of the index-coded samples was performed on a cBot Cluster Generation System using a TruSeq PE Cluster Kit v3-cBot-HS (Illumina, San Diego, CA, USA) according to the manufacturer’s instructions. After cluster generation, the libraries were sequenced on an Illumina HiSeq 2500 platform and 125-bp long paired-end reads were generated.

### Transcriptome assembly

Clean data were obtained by removing reads containing adapters, reads containing over 10 % of poly(N), and low-quality reads (>50 % of the bases had Phred quality scores ≤ 10) from the raw data. The Phred score (Q20) and GC content of the clean data were calculated. All the downstream analyses were based on the high quality clean data. Goat reference genome and gene model annotation files were downloaded from NCBI database (CHIR_1.0, NCBI) [60]. Index of the reference genome was built using Bowtie v2.0.6 [61, 62] and paired-end clean reads were aligned to the reference genome using TopHat v2.0.14 [63]. The mapped reads from each library were assembled with Cufflinks v2.2.1 [64]. Cufflinks was run with ‘min-frags-per-transfrag = 0’ and‘–library-type fr-firststrand’, and other parameters were set as default.

### Filtering pipeline to identify multiple exon lncRNAs

We filtered the assembled novel transcripts from the different libraries to obtain putative lncRNAs following the steps in the pipeline (Fig. 1) as follows. (1) Single exon transcripts and transcripts < 200-bp long were removed. (2) The remaining transcripts that overlapped (>1 bp) with goat protein-coding genes were removed. (3) Transcripts that were likely to be assembly artifacts or PCR run-on fragments according to the class code annotated by cuffcompare [65] were removed. Among the cuffcompare classes, only transcripts annotated as “i”, “u”, and “x” representing novel intronic, intergenic, and antisense transcripts respectively, were retained. (4) The Coding Potential Calculator (CPC) [66], Coding-Non-Coding-Index (CNCI) [67], and phylogenetic codon substitution frequency (PhyloCSF) [68] tools were used to assess the coding potential of the remaining transcripts, and transcripts with CPC score > 0, CNCI score > 0, and PhyloCSF Max_score > 100 were removed. (5) The remaining transcripts that contained a known protein-coding domain were removed. To accomplish this, each transcript sequence was translated in all six reading frames, then HMMER was used to exclude transcripts with a translated protein sequence that had a significant hit in the Pfam (PfamA and PfamB) database, release 28.0 [69]. (6) The remaining transcripts that belonged to known classes of small RNAs (including snRNA, tRNAs, and miRNAs) were removed using Rfam release 12.0 [70].

### Expression analysis

Cuffdiff 2.1.1 [71] was used to calculate FPKM scores for the lncRNAs and coding genes in each library. Differentially expressed lncRNAs between any two libraries were identified by edgeR (release 3.2) [72]. We used a P-adjust < 0.01 and an absolute value of the |log_2_(fold change)| > 2 as the threshold to evaluate statistical significance of lncRNA expression differences. We analyzed the clusters obtained by systematic analysis for all differentially expressed lncRNAs in eleven libraries using the Heatmaps software package in R [73].

### Target gene prediction and functional enrichment analysis

*Cis*-acting lncRNAs target neighboring genes [74, 75]. We searched for coding genes 10-kb upstream and downstream of all the identified lncRNAs and then predicted their functional roles as follows. The names of the neighbor genes were used to form a gene list that was input into DAVID software [46] for GO analysis. A KEGG enrichment analysis of the predicted target genes was performed with KOBAS software [47] using a hypergeometric test. GO terms and KEGG pathways with *P* < 0.05 were considered significantly enriched.

### Co-expression analysis

We used the expression levels of the identified lncRNAs and the known protein-coding genes from the four different developmental stages to analyze the co-expression of lncRNAs and protein-coding genes. We calculated Pearson’s correlation coefficients between the expression levels of 1747 lncRNAs and 19,846 protein-coding transcripts with custom scripts (*r* > 0.95 or *r* < −0.95). Then, we performed a functional enrichment analysis of the candidate lncRNA target genes using DAVID and KOBAS software. Significance was calculated using the Expression Analysis Systematic Explorer (EASE) test method (*P*-value < 0.05 was considered significant).

### Validation of differentially expressed lncRNAs by qPCR

Primers for the lncRNAs and internal control genes (Additional file 10) were designed using Primer-BLAST (http://www.ncbi.nlm.nih.gov/tools/primer-blast/). The expression levels of the lncRNAs were normalized to *ACTB*, *YWHAZ*, and *HPRT1*. Total RNA was converted to cDNA using a PrimeScript™ RT Reagent Kit with gDNA Eraser (TAKARA, Dalian, China), with oligo(dT) and random hexamer primers included in the kit. The qPCR was performed according to the SYBR Premix Ex Taq™ II instructions (TAKARA). The reaction volume contained 10 μl SYBR Premix Ex Taq™ II, 0.8 μl of 10 μM forward and reverse primers, 1.6 μl template cDNA, and dH_2_O to a final volume of 20 μl. The reactions were performed on a CFX96 Real-Time PCR System (Bio-Rad, CA, USA) as follows: 95 °C for 2 min, followed by 39 cycles of 95 °C for 10 s, and 10 s at the Tm indicated in Additional file 10: Table S8. Melting curve analysis was performed from 65 to 95 °C with increments of 0.5 °C. Amplifications were performed in triplicate for each sample. Relative gene expression levels were calculated using the 2^-ΔΔCt^ method [76], and data were expressed as least square mean ± standard error of the mean (SEM).

### Statistical analyses

All data were analyzed using one-way analysis of variance (ANOVA) to test homogeneity of variances via Levene’s test followed by Student’s *t*-test analyses in SAS software version 9.0 (SAS, Cary, North Carolina, USA). The significance level for the statistical analysis was *P* < 0.05.

## Additional files

Additional file 1: Table S1. Summary of RNA-seq data and reads mapped to the *Capra hircus* reference genome. (XLSX 13 kb) Additional file 2: Table S2. List of lncRNAs identified in the goat skeletal muscle libraries. (XLSX 171 kb) Additional file 3: Figure S1. Chromosome distribution of lncRNAs identified in goat skeletal muscle. (TIF 120 kb) Additional file 4: Table S3. List of differentially expressed lncRNAs from four skeletal muscle developmental stages. (XLSX 122 kb) Additional file 5: Figure S2. Hierarchical clustering analysis of all expressed lncRNAs from 11 libraries. (TIF 124 kb) Additional file 6: Table S4. Protein-coding genes detected 10-kb upstream and downstream of the differentially expressed lncRNAs. (XLSX 149 kb) Additional file 7: Table S5. Functional enrichment analysis of protein-coding genes targeted by *cis*-acting lncRNAs. (XLSX 47 kb) Additional file 8: Table S6. Co-expression analysis between protein-coding genes and lncRNAs (Pearson’s correlation coefficients > 0.95 and < −0.95). (XLSX 16453 kb) Additional file 9: Table S7. Functional enrichment analysis of protein-coding genes co-expressed with lncRNAs. (XLSX 160 kb) Additional file 10: Table S8. Primers used in the qPCR analysis. (XLSX 9 kb) Additional file 11: The transcript annotation information for the lncRNAs. (GTF 2080 kb)
