# Supplementary figures and images for: Genome-wide identification and characterization of long non-coding RNAs in developmental skeletal muscle of fetal goat
Source: BMC Genomics. 2016 Aug 22;17(1):666. doi: 10.1186/s12864-016-3009-3 (PMC4994410; doi:10.1186/s12864-016-3009-3)

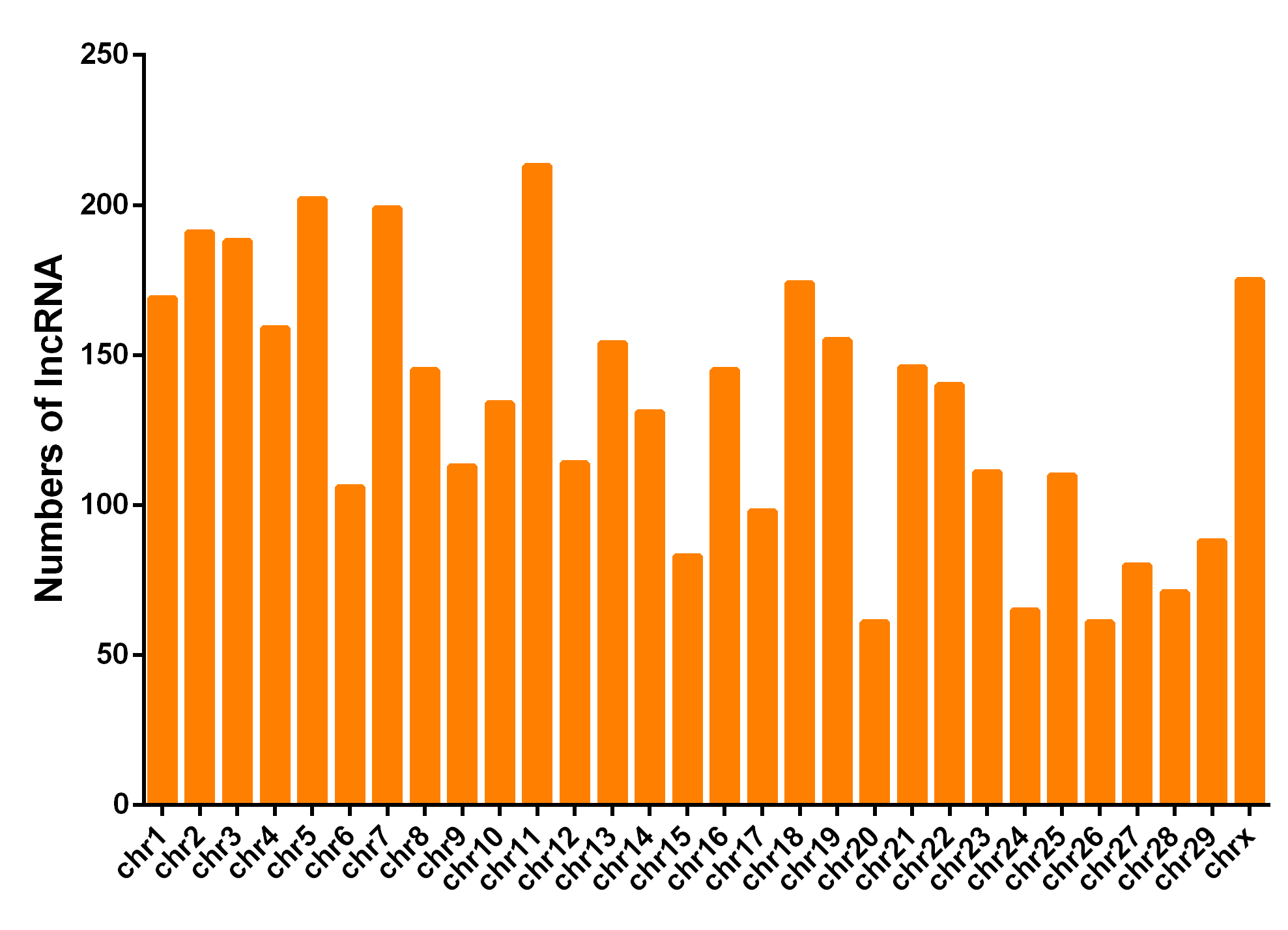

Supplement: Additional file 3: Figure S1. — Chromosome distribution of lncRNAs identified in goat skeletal muscle. (TIF 120 kb) [file 12864_2016_3009_MOESM3_ESM.tif]

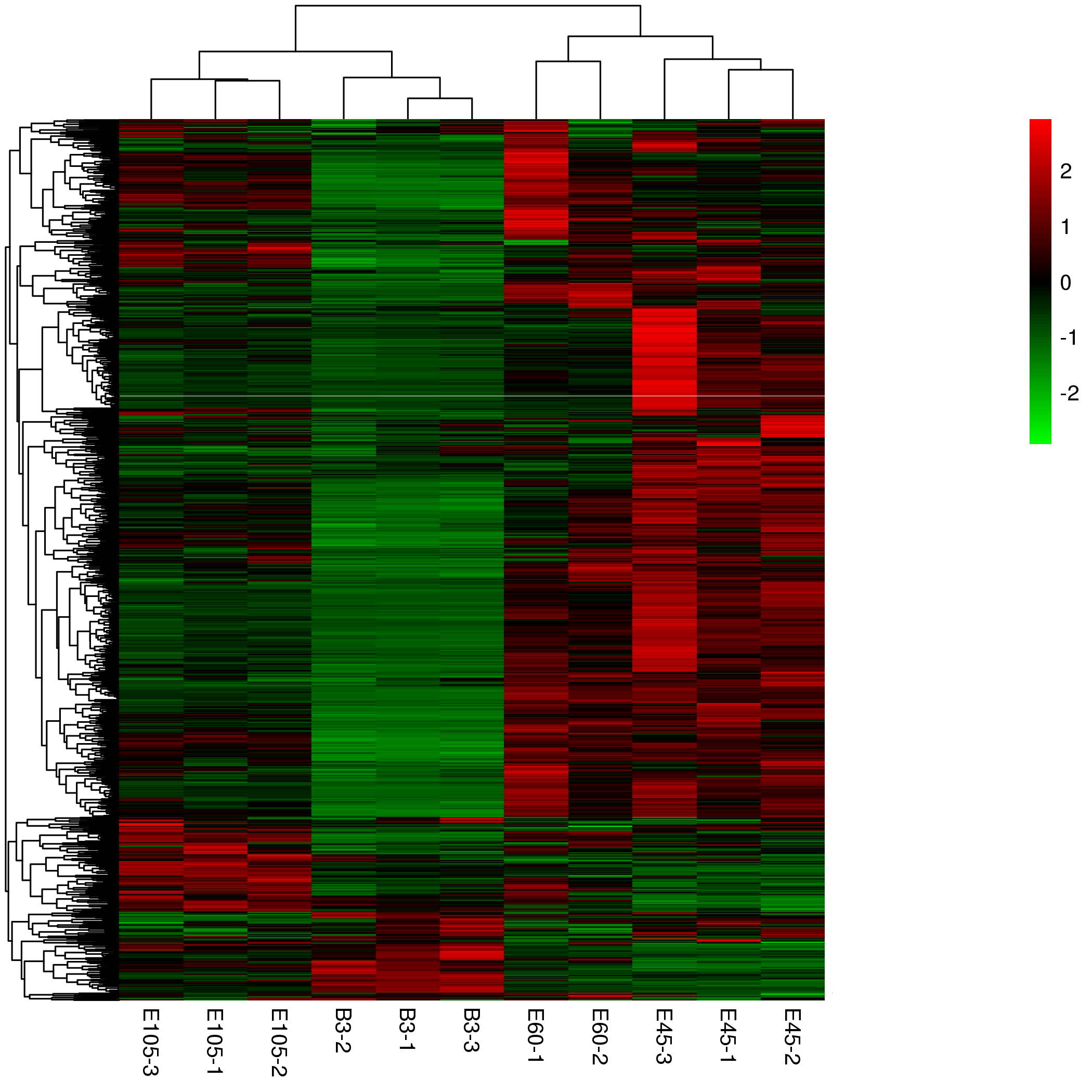

Supplement: Additional file 5: Figure S2. — Hierarchical clustering analysis of all expressed lncRNAs from 11 libraries. (TIF 124 kb) [file 12864_2016_3009_MOESM5_ESM.tif]
